# QTL Identification for Cooking and Eating Quality in *indica* Rice Using Multi-Parent Advanced Generation Intercross (MAGIC) Population

**DOI:** 10.3389/fpls.2018.00868

**Published:** 2018-07-10

**Authors:** Kimberly S. Ponce, Guoyou Ye, Xiangqian Zhao

**Affiliations:** ^1^Rice Breeding Platform, International Rice Research Institute, Los Baños, Philippines; ^2^CAAS-IRRI Joint Laboratory for Genomics-Assisted Germplasm Enhancement, Agricultural Genomics Institute, Shenzhen, China; ^3^Institute of Crop Science and Nuclear Technology Utilization, Zhejiang Academy of Agricultural Science, Hangzhou, China

**Keywords:** cooking quality, eating quality, MAGIC population, association mapping, rice

## Abstract

Association mapping using a multi-parent advanced generation intercross (MAGIC) population provides a promising tool in genetic dissection of rice cooking and eating quality (CEQ). In this study, QTLs were identified for ten physicochemical properties related to CEQ using 508 F_6_ MAGIC lines. The whole population and eight founder lines were genotyped with 6K Illumina Infinium HD Assay. All traits had high heritability estimates and showed a large genetic variation in the MAGIC population. Highly significant phenotypic correlations were present between traits. AC was significantly positively correlated with PKT, TV, FV, SBV, PKT, and RT but significantly negatively correlated with GC and BDV. Seventeen QTLs were identified for all traits. GBSSI locus was hosted or closely to nine QTLs, *qAC6*, *qGC6.1*, *qPKT6.1*, *qPKV6*, *qBDV6.1*, *qTV6.1*, *qFV6*, *qSBV6*, and *qRT6*, suggesting that GBSSI impacts the overall CEQ. Another locus closed to SSIIa, located at 6.99 Mb, affects five traits, GC, PKT, BDV, SBV, and PT. The identified QTLs revealed small to modest effects where the highest percentage of phenotypic variance explained was 17.18%. These QTLs are directly relevant and useful in breeding for CEQ in *indica* rice. These results also confirmed that QTL mapping via association mapping using a MAGIC population is a powerful method in genetic analysis of complex traits.

## Introduction

Grain quality is one of the most important quantitative traits in rice. It is a multi-faceted trait involving physical and biochemical aspects relating to milling, appearance, cooking and eating quality (CEQ), and nutrition (He et al., 1999; Fitzgerald et al., 2009; Bao, 2014). Among these, CEQ is the most important aspect especially in consumers’ perspective serving as the basis of choice for quality rice. CEQ mainly affects the easiness of cooking, firmness, and cohesiveness of cooked rice and CEQ is directly related to four physicochemical properties, the amylose content (AC), gel consistency (GC), gelatinization temperature (GT), and pasting viscosity (PV) (Dela Cruz and Khush, 2000; Fitzgerald et al., 2009; Bao, 2014). Hence, most of the genetics studies use these traits as rice quality parameters.

Amylose content is the most important chemical property affecting CEQ, which is considered as a good indicator of the appearance and texture of cooked rice (Lanceras et al., 2000). GC is a measure of firmness and stickiness of cooked rice upon cooling and therefore, a measure of strength of the gel. It is used as one of the key parameters to determine the textural property of cooked rice (Cuevas and Fitzgerald, 2012). It is also used to differentiate rice varieties having similar AC class, since GC is highly associated with AC (i.e., rice variety with low AC tend to have high GC or soft gel upon cooling) (Dela Cruz and Khush, 2000; Cuevas and Fitzgerald, 2012). Thus, GC is negatively correlated with AC. Pasting viscosity is widely used to assess starch properties in rice (Bao and Xia, 1999; Yan et al., 2005; Allahgholipour et al., 2006). It is measured using rapid visco analyzer (RVA), a rotational viscometer, which mimics the cooking process (Bao, 2014). To some extent, PV is interchangeably used with gelatinization but the two parameters are different. Gelatinization specifically refers to molecular disintegration of starch molecules, whereas PV refers to starch viscosity development. This suggests that PV occurs after gelatinization.

Several studies have reported major genes/QTLs for CEQ in rice using DH population (He et al., 1999; Bao et al., 2000), RILs (Bao et al., 2003), and CSSLs (Zhang et al., 2013) via bi-parental linkage mapping. Among these genes identified, it is well known that GBSSI gene is the major gene responsible for CEQ (He et al., 1999; Tran et al., 2011). Most of these QTLs detected had very high phenotypic variance explained, however, these QTLs were often not transferable to other genetic backgrounds since the estimated effects are limited to the population studied (Holland, 2007). Hence, development of rice varieties with superior CEQ is confined to very few genetic backgrounds.

Genetic analysis via association mapping (AM) offers a great opportunity to identify significant marker-trait associations (MTAs) (Huang et al., 2012). AM is a practical application of linkage disequilibrium (LD) mapping since its principle lies in LD. Hence, LD greatly influence the resolution of mapping QTL associated with the trait under study. The use of natural accessions for AM results in high mapping resolution due to its great LD decay over time brought by years of numerous recombination. However, there is an extensive population structure and cryptic kinship in natural accessions which increases the chance of discovering false positive MTAs (Zhu et al., 2008; Huang et al., 2015). Multi-parent advanced generation intercross (MAGIC) population, an extension of advanced intercrossed lines (Darvasi and Soller, 1995), offers an alternative type of mapping population that overcomes the limitations of family-based mapping using populations derived from biparental cross and AM using natural accessions. It involves the use of multiple genetically diverse parents to come up with a population of high allelic and phenotypic diversity in combination with high levels of recombination events brought about by several cycles of inter-mating (Mackay and Powell, 2007; Cavanagh et al., 2008; Huang et al., 2015). Increased recombination events reduce the extent of LD and thus increase genetic mapping resolution (Huang et al., 2015). Furthermore, since the founder lines contributing to the allelic and phenotypic diversity are well known, the population structure and kinship are highly controlled. This suggests that MAGIC population offers a great potential for mapping complex quantitative traits by preventing spurious MTAs. The effective use of MAGIC population in QTL identification via AM has been conducted in several cereal crops such as rice (Bandillo et al., 2013; Meng et al., 2016, 2017), wheat (Mackay et al., 2014), and barley (Sannemann et al., 2015). Thus, the use of MAGIC population for genetic studies in crops has risen in the past years.

In this study, we aimed to identify genes/QTLs underlying CEQ in rice using MAGIC population using 6K Illumina Infinium HD Assay. Association analysis was conducted by employing mixed linear model (MLM) to identify the loci associated with CEQ. The results will be valuable in breeding *indica* rice to improved CEQ and for further use of MAGIC population to illuminate genetic basis of other grain quality traits.

## Materials and Methods

### Plant Material

The MAGIC population was developed at IRRI using eight diverse founder lines as described in detail by Meng et al. (2016, 2017). The eight founder lines including SAGC-08 (A), HHZ5-SAL9-Y3-Y1 (B), BP1976B-2-3-7-TB-1-1 (C), PR 33282-B-8-1-1-1-1-1 (D), FFZ1 (E), CT 16658-5-2-2SR-2-3-6MP (F), IR 68 (G), and IR 02A127 (H) were selected based on genetic relationship and relevance to rice breeding in terms of yield, grain quality, and biotic or abiotic stress tolerance (Supplementary Table 1). A total of 532 F_6_ lines randomly chosen from the population were used in this study.

### Phenotyping

The field trials were conducted at the headquarters of International Rice Research Institute (IRRI), Los Baños, Laguna, Philippines in 2014 wet season. Trials design and field management were described by Meng et al. (2016). One hundred and six lines (One-fifth of 532 F_6_ lines) and the eight parental lines were replicated three times. Seeds were sown in the seedling nursery on 26 May, 2014. Twenty-day old seedlings were transplanted into field of six-by-six plots with a total of 36 plants in each plot and with a spacing distance of 20 cm × 20 cm. An augmented randomized complete block design was used to layout the trials. A total of 16 plants from middle rows were harvested. Finally, 508 lines yielded enough paddies were selected for further grain quality analysis. Freshly harvested paddy was dried to moisture content of 12–14% and equilibrated in paper bags at room temperature for 2 months. Physicochemical traits of parents and MAGIC population were evaluated at Grain Quality and Nutrition Services Laboratory, IRRI. AC was measured by standard iodine colorimetry method described in ISO 6647-2-2011. GC was measured based on the method described by Cagampang et al. (1973). Pasting viscosity (PV) parameters, the peak viscosity (PKV), trough viscosity (TV), breakdown viscosity (BDV), final viscosity (FV), setback viscosity (SBV), peak time (PKT), pasting temperature (PT), and retrogradation (RT) were measured by RVA to evaluate rheological properties of starch structure (RVA 4500, Perten, Sweden) based on the manufacturer’s instructions. AC, GC, and PV parameters were measured in three replicates.

### DNA Extraction and SNP Genotyping

High-throughput DNA extraction technique was carried out at the IRRI Genotyping Service Laboratory using sbeadex^®^ mini plant kit. High quality DNA samples were quantified with Quant-iT^TM^ PicoGreen^®^ dsDNA Assay Kit. SNP Genotyping was performed using 6K Illumina Infinium HD Assay (Illumina Inc., San Diego, CA, United States) following manufacturer’s protocol. Automated calling of SNP array data was done using Illumina GenomeStudio software. A total of 4695 SNP markers yielded good quality genotypic calls. A stringent filtering strategy was further conducted to choose high quality SNPs for association and linkage mapping. All heterozygous markers were set to missing. Markers with minor allele frequency (<0.03) and with missing values (≥0.1) were removed. Highly correlated markers (*r*^2^ > 0.95) were also excluded from the SNP data set using custom R scripts.

### Statistical Analysis

Adjusted trait values for each RIL were obtained using the PBTools software developed by IRRI ^1^. The broad sense heritability ($H_{\text{B}}^{2}$) of each trait was calculated using the following equation, $H_{\text{B}}^{2}$ = σ_G_/(σ_G_ + σ_E_/1.2), where σ_G_,σ_E_ and 1.2 represent estimate genotypic variance, experimental error variances, and the number of replication, respectively. Pairwise correlation coefficients for all the traits were estimated using the STAR software (see footnote 1).

Analysis of population structure, LD, and MTA mapping were carried out as described by Meng et al. (2016, 2017). MLM with population structure and familial relatedness between genotypes was conducted using TASSEL V5.0 (Bradbury et al., 2007). Significant MTAs were identified based on their significant association threshold (positive false discovery rate, *q^FDR^*< 0.05) (Benjamini and Hochberg, 1995). The *q*-values were calculated using the QVALUE in R (Storey and Tibshirani, 2003). Peaks exhibiting the significance threshold level within a physical distance of 1.25 Mb were considered as a single QTL (Meng et al., 2016, 2017). R^2^ was represented explained phenotypic variation using TASSEL V5.0. The plots were visualized using *qqman* package in R.

## Results

### Phenotypic Variation

PR 33282-B-8-1-1-1-1-1 had the lowest AC (0.8%) among the founder lines, therefore considered as glutinous parent (**Table 1**). The rest of the founder lines were non-waxy. SAGC-08, FFZ1, and IR 02A127 were under low AC class (10–20%) while CT 16658-5-2-2SR-2-3-6MP, BP1976B-2-3-7-TB-1-1, HHZ 5-SAL 9-Y 3-Y 1, and IR 68 were under intermediate AC class (20–25%). There were 232, 235, and 12 MAGIC lines with low, intermediate, and high AC classes, respectively (Supplementary Table 2). In addition, 29 lines were glutinous. The average AC of MAGIC population was 17.9%.

**Table 1:** Characteristics and broad sense heritability for cooking and eating quality traits of parents and MAGIC population.

Traits^a^	Parents^b^								MAGIC population		
	A	B	C	D	E	F	G	H	Mean ±*SD*	Range	$H_{\text{B}}^{2}$^c^
AC (%)	11.6	20.8	20.7	0.8	14.6	20	23.3	17.6	17.9 ± 5.8	0–27.2	0.95
GC (mm)	51.0	50.5	61.5	90.5	57.0	40.5	21.5	58.0	50.2 ± 20.9	20–100	0.81
PKV (RVU)	3061	3078	3012	2000	3183	2723	3134.5	3014.5	2908.9 ± 519	515–4010	0.87
TV (RVU)	1588.5	1498	1547.5	1099.5	1790.5	1603	2431.5	1683.5	1848.6 ± 468.0	364–3183	0.88
BDV (RVU)	1472.5	1580	1464.5	900.5	1392.5	1120	703	1331	1038.4 ± 427.0	45–2202	0.92
FV (RVU)	3125.5	3361	3135.5	1350.5	3561.5	3912	5625.5	3708	3857.2 ± 1060.6	488–6904	0.90
SBV (RVU)	64.5	283	123.5	−649.5	378.5	1189	2491	693.5	970.2 ± 946.0	−1101–3395	0.90
PKT (min)	6.1	5.8	5.9	4.0	6.2	6.1	6.4	6.0	6.1 ± 0.5	3.7–7.0	0.94
PT (°C)	71.7	77.5	77.5	68.5	72.5	78.7	70.5	77.2	74.4 ± 3.7	66.5–84.5	0.84
RT (RVU)	1537	1863	1588	251	1771	2309	3194	2024.5	2008.6 ± 749.6	121–4163	0.85

*^a^Abbreviations: AC, amylose content; GC, gel consistency; PKV, peak viscosity; TV, trough viscosity; BDV, breakdown viscosity; FV, final viscosity; SBV, setback viscosity; PKT, peak time; PT, pasting temperature; RT, retrogradation. ^b^A–H represents eight founder liens, SAGC-08 (A), HHZ5-SAL9-Y3-Y1 (B), BP1976B-2-3-7-TB-1-1 (C), PR 33282-B-8-1-1-1-1-1 (D), FFZ1 (E), CT 16658-5-2-2SR-2-3-6MP (F), IR 68 (G), and IR 02A127 (H), respectively. ^c^$H_{B}^{2}$: broad sense heritability estimate.*

The GC of PR 33282-B-8-1-1-1-1-1 was the highest (**Table 1**) among founder lines suggesting its soft textural property. Similarly, BP1976B-2-3-7-TB-1-1 had the soft textural property with a mean value of (61.5 mm). HHZ 5-SAL 9-Y 3-Y 1, SAGC-08, FFZ1, CT 16658-5-2-2SR-2-3-6MP, and IR 02A127 were under medium GC class, therefore had flaky textural property. IR 68 had a hard GC class suggesting very flaky textural property. A total of 190, 145, and 173 MAGIC lines with high, intermediate, and low GC, respectively (Supplementary Table 2). The average GC of the whole population was 50.2 mm.

The RVA was used to mimic the cooking process in rice. In the process, viscosity increases reaching its maximum in the first phase called the PKV [6, 29]. This phase is characterized by swelling and paste formation as the rice starch is being heated up to 65°C with water. PKV of PR 33282-B-8-1-1-1-1-1 was the lowest among founder lines (**Table 1**), since this line was glutinous with glossy sticky firm and soft textural property. The other founder lines had almost the same PKV values and were much higher than PR 33282-B-8-1-1-1-1-1. This suggests rice with soft textural property will tend to swell and burst faster than those with hard and very flaky textural property. Average PKV of the MAGIC population was 2910. PKT is the time required to reach PKV hence low PKV is indicative of low PKT. The founder line PR 33282-B-8-1-1-1-1-1, with the lowest PKV, had also the lowest PKT values (**Table 1**). The PKT values of MAGIC population ranged from 3.7 to 7 min with a mean of 6.1 min. Similar with PKT, TV, FV, SBV, PT, and RT of PR 33282-B-8-1-1-1-1-1 are the lowest among founder lines (**Table 1**). On the contrary, except for BDV, the remains of other seven RVA properties of IR 68 are the highest among eight parents (**Table 1**).

Broad sense heritability was estimated for all the traits (**Table 1**). Results showed that all the physicochemical traits had high heritability estimates ranging from 0.81 to 0.95. Notably, AC had the highest heritability and therefore the trait could be easily passed onto the progeny. The high heritability estimates for the other traits were not surprising since AC mainly influences the overall CEQ traits.

### Trait Correlations

Based on Pearson’s correlation analysis between physicochemical parameters, almost all the traits were significantly correlated (**Table 2**). AC was significantly positively correlated with PKT, TV, FV, SBV, PKT, and RT but significantly negative correlated with GC and BDV, which indicate that disruption of starch molecules become harder as AC increase. These findings supported the fact that indeed AC is the most important factor affecting cooking and eating properties. In addition, the correlation coefficient between BDV and other traits analyzed was strongly significant as well. BDV had positive correlation with GC, PKV, and PT, while it was negatively correlated with other traits. It is also noteworthy that PT only shows significant correlation with three traits (AC, PKV, and BDV) with relatively low coefficient.

**Table 2:** Pairwise correlation values among ten physicochemical properties of MAGIC population.

Traits^a^	GC	PKV	TV	BDV	FV	SBV	PKT	PT	RT
**AC**	−0.6267^∗∗b^	0.2623^∗∗^	0.5632^∗∗^	−0.2984^∗∗^	0.8034^∗∗^	0.7568^∗∗^	0.6710^∗∗^	0.1173^∗∗^	0.7851^∗∗^
**GC**		−0.0146	−0.4393^∗∗^	0.4638^∗∗^	−0.6852^∗∗^	−0.7602^∗∗^	−0.5843^∗∗^	0.0641	−0.6958^∗∗^
**PKV**			0.6301^∗∗^	0.5250^∗∗^	0.4536^∗∗^	−0.0401	0.3682^∗∗^	0.1740^∗∗^	0.2484^∗∗^
**TV**				−0.3301^∗∗^	0.7878^∗∗^	0.5375^∗∗^	0.5968^∗∗^	−0.0317	0.4904^∗∗^
**BDV**					−0.3121^∗∗^	−0.6379^∗∗^	−0.2065^∗∗^	0.2462^∗∗^	−0.2355^∗∗^
**FV**						0.8723^∗∗^	0.6452^∗∗^	0.0656	0.9231^∗∗^
**SBV**							0.5214^∗∗^	−0.0219	0.8986^∗∗^
**PKT**								0.0717	0.5404^∗∗^
**PT**									0.1183^∗∗^

*^a^Abbreviations: described in **Table 1**. ^b∗∗^Represents that p-value is lower than 0.01.*

### QTLs Identified by Association Analysis

**FIGURE 1:**
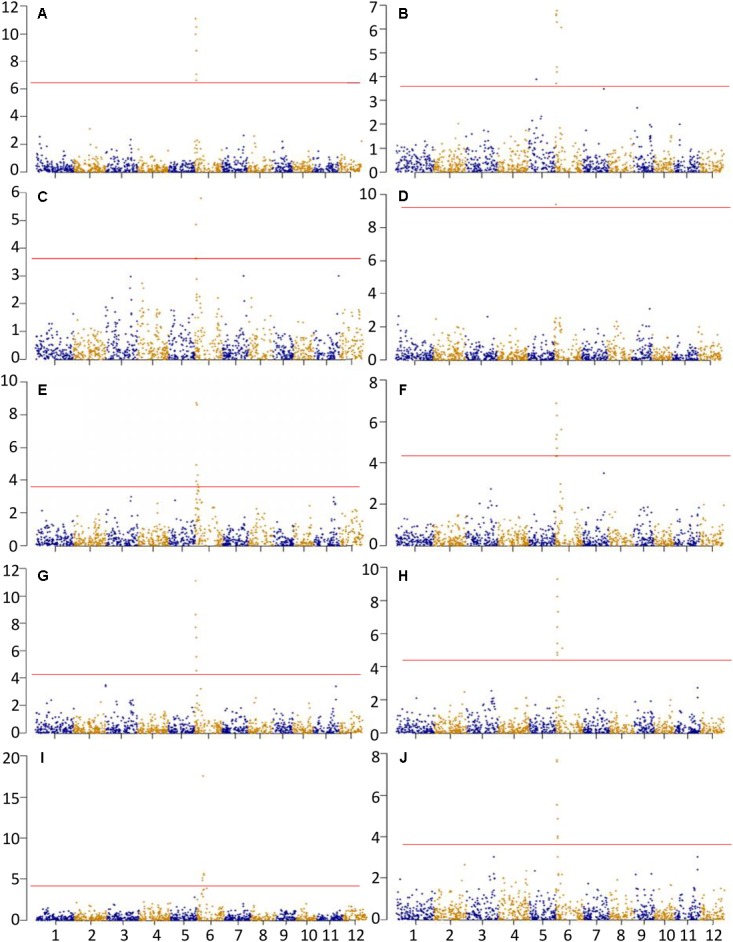
Association analysis for **(A)** AC, **(B)** GC, **(C)** PKT, **(D)** PKV, **(E)** TV, **(F)** BDV, **(G)** FV, **(H)** SBV, **(I)** PT, and **(J)** RT. *X*-axis and *Y*-axis represent chromosome and negative log (*q*-value), respectively. Horizontal solid line indicates the significant threshold *P* < 0.05.

A total of 62 significant MTAs were identified for the ten physicochemical traits related to CEQ. The Manhattan plots for all the traits were presented in **Figure 1**. The details of MTAs were given in Supplementary Table 3. A total of 17 QTLs were identified by delineating significant MTAs within a physical distance of 2.5 Mb into a single QTL (**Table 3**).

**Table 3:** Putative QTLs for cooking and eating quality.

Traits^a^	QTL	Marker	Chr^b^	Pos^c^	Interval (NM^d^)	Potential Gene	*q*-value	%*R*^2e^	Allele	Effect
AC	*qAC6*	id6000911	6	1378891	1.38–2.13 (7)	GBSSI	0.0000	13.49	A/G	−4.65
GC	*qGC5*	id5004295	5	8384781	8.38 (1)		0.0294	3.14	A/G	−10.27
GC	*qGC6.1*	5883472	6	2130189	1.38–2.13 (7)	GBSSI	0.0001	5.96	A/G	−14.27
GC	*qGC6.2*	6026032	6	6990453	6.99 (1)	SSIIa	0.0004	5.24	C/T	12.89
PKT	*qPKT6.1*	id6000911	6	1378891	1.38–1.50 (2)	GBSSI	0.0044	4.03	A/G	−0.17
PKT	*qPKT6.2*	6026032	6	6990453	6.99 (1)	SSIIa	0.0008	4.95	C/T	−0.18
PKV	*qPKV6*	fd8	6	1768006	1.77 (1)	GBSSI	0.0000	8.51	A/C	403.24
TV	*qTV6*	id6000911	6	1378891	1.38–2.43 (6)	GBSSI	0.0000	7.69	A/G	−277.62
BDV	*qBDV6.1*	id6000911	6	1378891	1.38–2.13 (7)	GBSSI	0.0001	6.01	A/G	336.61
BDV	*qBDV6.2*	6026032	6	6990453	6.99 (1)	SSIIa	0.0011	4.78	C/T	273.59
FV	*qFV6*	id6000911	6	1378891	1.38–2.13 (6)	GBSSI	0.0000	10.34	A/G	−800.88
SBV	*qSBV6.1*	id6000911	6	1378891	1.38–2.13 (7)	GBSSI	0.0000	8.40	A/G	−839.72
SBV	*qSBV6.2*	6026032	6	6990453	6.99 (1)	SSIIa	0.0028	4.25	C/T	−543.11
PT	*qPT6.1*	5980679	6	5707236	5.71–6.40(3)		0.0023	4.33	A/G	1.91
PT	*qPT6.2*	6026032	6	6990453	6.99–7.63(4)	SSIIa	0.0000	17.18	C/T	4.07
PT	*qPT6.3*	SNP-6_10761128	6	10762128	10.76 (1)		0.0280	3.14	A/G	−1.92
RT	*qRT6*	id6000911	6	1378891	1.38–2.13 (6)	GBSSI	0.0000	6.85	A/G	−515.99

*^a^Abbreviations: Described in **Table 1**. ^b^Chromosome number. ^c^Physical position (bp). ^d^Number of significant markers within the QTL interval. ^e^Percentage of phenotypic variance explained by the marker.*

A total of seven significant MTAs were identified for AC, all of which were located on chromosome 6 (**Figure 1A** and Supplementary Table 3). These MTAs were located on the interval 1.38–2.13 Mb and with peak SNP at an A/G polymorphism. The allele G was associated with decreased AC, accounting 13.49% of total phenotypic variance (**Table 3**). The SNP marker, fd8, an A/C polymorphism was just located on GBSSI and explained 6.24% of the total phenotypic variance.

Three QTLs *qGC5*, *qGC6.1*, and *qGC6.2* were identified for GC from a total of nine significant MTAs (**Figure 1B** and Supplementary Table 3). These were located on chromosomes 5 and 6, and explained 3.14, 5.96, and 5.24% of the phenotypic variation, respectively (**Table 3**). The peaked SNPs for *qGC5* and *qGC6.1* were both A/G polymorphisms located at 8.38 and 2.13 Mb on chromosome 5 and 6, respectively, with the allele G increasing GC. The allele C in C/T polymorphism of *qGC6.2* increases GC. An A/C polymorphism was on GBSSI gene explained 3.63% of the total phenotypic variation. This result was consistent with previous study that GBSSI was a major gene affecting GC.

Two QTLs were identified for PKT from a total of three significant MTAs (**Table 3**, **Figure 1C** and Supplementary Table 3). The *qPKT6.1* and *qPKT6.2* were located on chromosome 6 within 1.38 to 1.50 Mb and at 6.99 Mb, explained 4.03 and 4.95% of the phenotypic variations, respectively. The *qPKT6.1* peaked at A/G polymorphism whereas *qPKT6.2* peaked at C/T polymorphism (**Table 3**).

One QTL (*qPKV6*) was identified for PKV (**Figure 1D** and Supplementary Table 3). This QTL was located on chromosome 6 at 1.77 Mb and peaked at A/C polymorphism (**Table 3**). The allele C was associated with decreased PKV, explained about 8.5% of phenotypic variation. Moreover, the peaked SNP corresponded with GBSSI gene.

A total of six significant MTAs were identified for TV on chromosome 6, which were delineated into one QTL, *qTV6* (**Figure 1E** and Supplementary Table 3). The *qTV6* was located within 1.38 to 2.87 Mb and peaked at A/G polymorphism with allele G being associated with decreased TV, explained 7.69% of the phenotypic variation (**Table 3**).

Two QTLs, *qBDV6.1* and *qBDV6.2*, both located on chromosome 6 were identified for BDV from a total of eight significant MTAs (**Figure 1F** and Supplementary Table 3). The *qBDV6.1* located within 1.38 to 2.15 Mb peaked at A/G polymorphism (**Table 3**), which co-localized with the GBSSI gene. The *qBDV6.2* peaked at C/T polymorphisms located at 6.99 Mb. The alleles G on *qBDV6.1* and allele C on *qBDV6.2* were associated with decreased BDV, accounting for 6.01 and 4.78% of phenotypic variation, respectively.

Six significant MTAs were identified for FV (**Figure 1G** and Supplementary Table 3). These MTAs were delineated into a single QTL, the *qFV6* (**Table 3**). The QTL was located in the interval 1.38 to 2.15 Mb on chromosome 6. Moreover, this peaked at A/G polymorphism and explained 10.34% of the total phenotypic variance. The allele G in this SNP was associated with decreased FV.

A total of two QTLs *qSBV6.1* and *qSBV6.2* were identified for SBV from eight significant MTAs (**Figure 1H** and Supplementary Table 3). The *qSBV6.1* located within 1.38 to 2.13 Mb on chromosome 6 peaked at A/G polymorphism. The *qSBV6.2* on the same chromosome peaked at C/T polymorphism and was located on the same chromosome at 6.99 Mb (**Table 3**). The alleles G and C of the two peaked SNPs were associated with decreased SBV.

Eight significant MTAs were identified for PT and delineated into three QTILs, the *qPT6.1*, *qPT6.2*, and *qPT6.3* (**Figure 1I** and Supplementary Table 3). The *qPT6.1* located within 5.71 to 6.40 Mb peaked at the A/G polymorphism and with allele A associated with increased PT, explained 4.33% of the total phenotypic variance. The *qPT6.2* located within 6.99 to 7.43 Mb peaked at the C/T polymorphism and with allele C associated with decreased PT, explained 17.18% of the total phenotypic variance. The *qPT6.3* peaked at A/G polymorphism, and explained 3.14% of the phenotypic variance (**Table 3**).

Six significant MTAs were identified for RT and delineated into a single QTL *qRT6* (**Figure 1J** and Supplementary Table 3). The QTL was located on chromosome 6 within 1.38 to 2.13 Mb, accounting for 6.85% phenotypic variation. The peaked SNP, A/G polymorphism, in the region was located at 1.38 Mb and with allele G associated with decreased RT.

## Discussion

### Pleiotropic Effects and Significant Correlations Among Traits

Pleiotropy is common phenomena in plant genetics. In this study, one QTL locus was hosted or closely to nine QTLs, *qAC6*, *qGC6.1*, *qPKT6.1*, *qPKV6*, *qBDV6.1*, *qTV6.1*, *qFV6*, *qSBV6*, and *qRT6*, suggesting that this locus impact mostly characteristics of CEQ in rice (**Figure 1** and **Table 3**). Specifically, the SNP marker fd8, an A/C polymorphism, located at a physical distance of 1.77 Mb corresponded to GBSSI gene. Another QTL locus, closed to SSIIa, anchored at 6.99 Mb affecting five traits, GC, PKT, BDV, SBV, and PT. These results were coincident with other studies (Lanceras et al., 2000; Wan et al., 2004; Ebadi et al., 2013; Bao, 2014), indicating pleiotropic effects are very common traits related to rice quality. Pleiotropic effects are important in crop improvement especially when QTL/gene affects multiple unrelated traits (i.e., desirable and undesirable). In addition, AC was negatively correlated with GC and BDV (**Table 2**). Positive correlation of AC with all the RVA parameters except BDV indicates that high AC increase disruption of starch molecules resulting to harder cooked rice gel. Negative correlation of BDV with all RVA parameters especially with SBV indicates that starch remains undisrupted when BDV is low. Above results suggest glossy sticky and soft rice texture tend to undergo fast breakdown of starch molecules (Yan et al., 2005; Wang et al., 2007). These findings supported the fact that indeed AC is the most important factor affecting CEQ (Dela Cruz and Khush, 2000; Fitzgerald et al., 2009; Ni et al., 2011). In the present study, the QTLs exhibiting pleiotropy could be directly utilized for improving CEQ traits in *indica* rice, since the traits it affects were highly correlated.

### QTLs With Small Effects

In the present study, the best MTAs revealed only modest *R^2^* value suggesting low variance predicted by each SNP marker. The *qPT6.2* revealed only 17.18% of the phenotypic variance whereas the same QTL explained 88% variation in QTL mapping using RILs derived from *indica*-*japonica* cross (Wang et al., 2007). Moreover, candidate gene association analysis showed higher phenotypic variance explained for AC (Kharabian-Masouleh et al., 2012; Yang et al., 2014; Zhao et al., 2015). Many association analyses in plants reported low to modest *R^2^* value. Bandillo et al. (2013) and Qiu et al. (2015) reported *R^2^* values ranging from 11 to 20% and 6.6 to 22.2% for the several quantitative traits in rice, respectively. These corresponded with our results where *R^2^* values ranged from 2.3 to 17.2% (**Table 3**). Possible reasons for these includes insufficient marker coverage used in the study where the causal variant is not in perfect LD with the genotype thereby reducing the association power and variation explained by each SNP marker. This problem could be circumvented with the use of populations showing greater levels of LD (Sorkheh et al., 2008) and more marker coverage. Covering the genome with high marker density is now possible with increasing availability and great reduction in the cost of high throughput SNP genotyping platforms such as genotype-by-sequencing (GBS) (Elshire et al., 2011), Rice 44K (Zhao et al., 2011), and Rice 700K platforms (McCouch et al., 2016). The population size is another consideration to increase the variation explained by the SNP marker. In our study, the population size (508 lines) used is enough to capture high mapping power and resolution since it was suggested that at least 50 individuals per founder lines should be the size of MAGIC population (Cavanagh et al., 2008). However, it was not able to significantly increase the variation explained by best MTAs due to low marker coverage used in the study.

### Potential of MAGIC Population for Gene Discovery and Breeding

The use of MAGIC population for gene discovery and breeding are becoming more attractive for geneticists and breeders. Genetic diversity is paramount to crop improvement. Notably, MAGIC population covers high level of recombination which allows for wide allelic and phenotypic diversity and increased precision of QTL mapping (Cavanagh et al., 2008). More recombination events allow for the development of alien diversity resource for use in conventional breeding and conduit for genetic analysis studies. Moreover high throughput SNP genotyping platforms are becoming cheap and statistical approaches for such populations are now available. The MAGIC population used in this study showed wider phenotypic variation in almost all traits than the eight founder lines (**Table 1**), which suggested the formation of transgressive segregants on both directions. This offers breeders a wide variability to select superior lines to further develop varieties with improved CEQ. The MAGIC lines were presented in a matrix based on AC and GC as these two parameters were the most important factors determining CEQ (Supplementary Table 2). It is interesting that the population can be classified into ten subsets. Compared with eight parents, only four groups, the low AC-soft GC, low AC-hard GC, high AC-medium GC, and high AC-hard GC, were created during three times of intermating and several times of selfing. It is indicating that it is possible to select specific recombinant of AC and GC for different CEQ using MAGIC population. Currently, the MAGIC population we used in this study was part of population improvement program via recurrent selection in IRRI to develop elite varieties of good performance. Interestingly, MAGIC lines were also included in multi-environment yield trials in South Asia, South-east Asia, and Africa. A total of 21 high yielding lines with satisfactory agronomic performance were selected by IRRI breeders for further testing together with 700 lines developed from other IRRI breeding programs. Continuous evaluation of MAGIC lines for biotic and abiotic stresses would allow the development of outstanding lines with high yield and satisfactory agronomic performance under stress conditions. At the present situation, some of the MAGIC lines used in this study were part of salinity tolerance and nutrient deficiency experiments in IRRI.

## Conclusion

Overall our results provided details on the chances of QTL detection via association mapping in *indica* rice using MAGIC population. A total of 17 QTLs were detected for all the physicochemical traits using Infinium 6K genotyping platform, of which 9 and 5 QTLs corresponded to GBSSI and SSIIa genes, respectively. This supports previous studies that GBSSI and SSIIa genes mainly influence the overall CEQ. The QTLs identified revealed small to modest phenotypic variance ranging from 2.89 to 17.18%. These QTLs and the markers highly associated with the traits under study may be useful for breeding *indica* rice to improve CEQ. Moreover, these findings provided a direct support on the use of MAGIC population as valuable resource for detecting QTL via association mapping and for practical breeding.

## Author Contributions

XZ and GY designed the experiment. KP and XZ performed all the phenotypic evaluation, data analysis, and drafted the manuscript. All authors revised the paper and approved the final version to be published.

## Conflict of Interest Statement

The authors declare that the research was conducted in the absence of any commercial or financial relationships that could be construed as a potential conflict of interest.

## Footnotes

1 bbi.irri.org
