# Supplementary material for: QTL Identification for Cooking and Eating Quality in indica Rice Using Multi-Parent Advanced Generation Intercross (MAGIC) Population
Source: Front Plant Sci. 2018 Jul 10;9:868. doi: 10.3389/fpls.2018.00868 (PMC6048290; doi:10.3389/fpls.2018.00868)
Supplement: Supplementary file 1 [file Table_1.DOCX]

Supplementary Material

**QTL identification for cooking and eating quality in *indica* rice using multi-parent advanced generation intercross (MAGIC) population**

Kimberly S. Ponce, Guoyou Ye, Xiangqian Zhao^*^

^*^**Correspondence**: Dr. Xiangqian Zhao: [ricechina@hotmail.com](mailto:ricechina@hotmail.com)

# Supplementary Figures and Tables

**Supplementary table 1.** Characteristics of Founder Parents of the MAGIC Population

| **Parents** | **Code** | **GID** | **Origin** | **Characteristics** |
| --- | --- | --- | --- | --- |
| SAGC-08 | A | 3301214 | China | Short grain, thickness stem, drought tolerance |
| HHZ 5-SAL 9-Y 3-Y 1 | B | 2857043 | IRRI-GSR | Long grain, salt tolerant, good grain quality |
| BP1976B-2-3-7-TB-1-1 | C | 3300964 | Indonesia | Long grain, blast disease resistance |
| PR 33282-B-8-1-1-1-1-1 | D | 2752173 | PhilRice | Long grain, high yielding |
| FFZ1 | E | 2731019 | China | Long grain, high yielding, good grain quality |
| CT 16658-5-2-2SR-2-3-6MP | F | 3300994 | CIAT | Long grain, high yielding |
| IR 68 | G | 63333 | IRRI | Long grain, large grain, thickness stem |
| IR 02A127 | H | 967269 | IRRI | Long grain, comprehensive disease resistance |

**Supplementary table 2.** Number of Lines in Each Subset Grouped based on AC and GC

|  | **waxy** | **Low (AC<20)** | **Intermediate (20≤AC<25)** | **High (AC≥25)** | **Subtotal** |
| --- | --- | --- | --- | --- | --- |
| **High (GC≥ 60 mm)** | 29 | 129 | 28 | 4 | 190 |
| **Intermediate (40≤GC<60)** |  | 72 | 69 | 4 | 145 |
| **Low (GC<40mm)** |  | 31 | 138 | 4 | 173 |
| **Subtotal** | 29 | 232 | 235 | 12 | 508 |

**Supplementary table 3. List of QTLs and significant MTAs identified using PK model**

| **Trait^a^** | **QTL** | **Marker** | **Chr^b^** | **Pos^c^** | **q value** | ***%R^2^*^d^** | **Alelle** | **Effect** |
| --- | --- | --- | --- | --- | --- | --- | --- | --- |
| AC | *qAC6* | id6000911 | 6 | 1378891 | 0.0000 | 13.49 | A/G | -4.65 |
|  |  | 5865517 | 6 | 1421311 | 0.0000 | 9.15 | A/C | -3.38 |
|  |  | SNP-6_1500959 | 6 | 1501961 | 0.0000 | 10.33 | A/G | -4.32 |
|  |  | fd8 | 6 | 1768006 | 0.0001 | 6.24 | A/C | -2.96 |
|  |  | rd6001726 | 6 | 1940946 | 0.0001 | 5.80 | G/T | 2.86 |
|  |  | 5882239 | 6 | 2025629 | 0.0000 | 9.72 | A/G | 3.55 |
|  |  | 5883472 | 6 | 2130189 | 0.0000 | 7.94 | A/G | 3.35 |
| GC | *qGC5* | id5004295 | 5 | 8384781 | 0.0294 | 3.14 | A/G | -10.27 |
|  | *qGC6.1* | id6000911 | 6 | 1378891 | 0.0001 | 5.75 | A/G | 14.04 |
|  |  | 5865517 | 6 | 1421311 | 0.0001 | 5.79 | A/C | 13.09 |
|  |  | SNP-6_1500959 | 6 | 1501961 | 0.0414 | 2.99 | A/G | 11.13 |
|  |  | fd8 | 6 | 1768006 | 0.0106 | 3.63 | A/C | 10.95 |
|  |  | rd6001726 | 6 | 1940946 | 0.0003 | 5.48 | G/T | -14.05 |
|  |  | 5882239 | 6 | 2025629 | 0.0157 | 3.44 | A/G | -11.02 |
|  |  | 5883472 | 6 | 2130189 | 0.0001 | 5.96 | A/G | -14.27 |
|  | *qGC6.2* | 6026032 | 6 | 6990453 | 0.0004 | 5.24 | C/T | 12.89 |
| PKT | *qPKT6.1* | id6000911 | 6 | 1378891 | 0.0044 | 4.03 | A/G | -0.17 |
|  |  | SNP-6_1500959 | 6 | 1501961 | 0.0497 | 2.89 | A/G | -0.15 |
|  | *qPKT6.2* | 6026032 | 6 | 6990453 | 0.0008 | 4.95 | C/T | -0.18 |
| PKV | *qPKV6* | fd8 | 6 | 1768006 | 0.0000 | 8.51 | A/C | 403.24 |
| TV | *qTV6.1* | id6000911 | 6 | 1378891 | 0.0000 | 7.69 | A/G | -277.62 |
|  |  | 5865517 | 6 | 1421311 | 0.0280 | 3.10 | A/C | -165.09 |
|  |  | SNP-6_1500959 | 6 | 1501961 | 0.0040 | 4.02 | A/G | -230.51 |
|  |  | 5882239 | 6 | 2025629 | 0.0000 | 7.56 | A/G | 267.29 |
|  |  | 5891296 | 6 | 2430760 | 0.0421 | 2.91 | C/T | 188.69 |
|  |  | 5903052 | 6 | 2871279 | 0.0132 | 3.44 | C/T | 207.56 |
| BDV | *qBDV6.1* | id6000911 | 6 | 1378891 | 0.0000 | 6.01 | A/G | 336.61 |
|  |  | 5865517 | 6 | 1421311 | 0.0128 | 3.54 | A/C | 234 |
|  |  | SNP-6_1500959 | 6 | 1501961 | 0.0026 | 4.33 | A/G | 316.84 |
|  |  | fd8 | 6 | 1768006 | 0.0128 | 3.54 | A/C | 233.48 |
|  |  | rd6001726 | 6 | 1940946 | 0.0017 | 4.53 | G/T | -293.54 |
|  |  | 5882239 | 6 | 2025629 | 0.0058 | 3.92 | A/G | -274.56 |
|  |  | 5883472 | 6 | 2130189 | 0.0003 | 5.43 | A/G | -316.45 |
|  | *qBDV6.2* | 6026032 | 6 | 6990453 | 0.0012 | 4.78 | C/T | 273.59 |
| FV | *qFV6* | id6000911 | 6 | 1378891 | 0.0000 | 10.34 | A/G | -800.88 |
|  |  | 5865517 | 6 | 1421311 | 0.0000 | 7.80 | A/C | -622.27 |
|  |  | SNP-6_1500959 | 6 | 1501961 | 0.0000 | 6.87 | A/G | -701.8 |
|  |  | rd6001726 | 6 | 1940946 | 0.0080 | 3.76 | G/T | 458.68 |
|  |  | 5882239 | 6 | 2025629 | 0.0001 | 6.13 | A/G | 559.02 |
|  |  | 5883472 | 6 | 2130189 | 0.0011 | 4.74 | A/G | 513.05 |
| SBV | *qSBV6.1* | id6000911 | 6 | 1378891 | 0.0000 | 8.40 | A/G | -839.72 |
|  |  | 5865517 | 6 | 1421311 | 0.0000 | 7.34 | A/C | -687.47 |
|  |  | SNP-6_1500959 | 6 | 1501961 | 0.0002 | 5.53 | A/G | -750.27 |
|  |  | fd8 | 6 | 1768006 | 0.0058 | 3.88 | A/C | -525.26 |
|  |  | rd6001726 | 6 | 1940946 | 0.0015 | 4.57 | G/T | 597.87 |
|  |  | 5882239 | 6 | 2025629 | 0.0043 | 4.04 | A/G | 568.63 |
|  |  | 5883472 | 6 | 2130189 | 0.0000 | 6.41 | A/G | 698.66 |
|  | *qSBV6.2* | 6026032 | 6 | 6990453 | 0.0028 | 4.25 | C/T | -543.11 |
| PT | *qPT6.1* | 5980679 | 6 | 5707236 | 0.0023 | 4.33 | A/G | 1.91 |
|  |  | 5987847 | 6 | 5894169 | 0.0043 | 4.01 | C/T | -1.84 |
|  |  | id6004038 | 6 | 6399722 | 0.0442 | 2.91 | A/C | 1.62 |
|  |  | 6026032 | 6 | 6990453 | 0.0000 | 17.18 | C/T | 4.07 |
|  |  | 6033802 | 6 | 7191499 | 0.0011 | 4.65 | G/T | -2.79 |
|  |  | 6037087 | 6 | 7259003 | 0.0009 | 4.82 | C/T | -2.86 |
|  |  | 6053591 | 6 | 7629011 | 0.0392 | 2.98 | G/T | 1.62 |
|  | *qPT6.2* | SNP-6_10761128 | 6 | 10762128 | 0.0280 | 3.14 | A/G | -1.92 |
| RT | *qRT6* | id6000911 | 6 | 1378891 | 0.0000 | 6.85 | A/G | -515.99 |
|  |  | 5865517 | 6 | 1421311 | 0.0000 | 6.77 | A/C | -461.6 |
|  |  | SNP-6_1500959 | 6 | 1501961 | 0.0011 | 4.73 | A/G | -459.39 |
|  |  | rd6001726 | 6 | 1940946 | 0.0280 | 3.17 | G/T | 341.18 |
|  |  | 5882239 | 6 | 2025629 | 0.0234 | 3.26 | A/G | 334.97 |
|  |  | 5883472 | 6 | 2130189 | 0.0043 | 4.06 | A/G | 380.55 |

^a^ Abbrev: Described in Table I.

^b^ Chromosome number.

^c^ Physical position (bp).

^d^ Number of significant markers within the QTL interval.

^e^ Percentage of phenotypic variance explained by the marker.
